# Suppressing Effect of Flavonoid Compounds on Lipids Photooxidation of Sheep Red Blood Cells and Oleic Acid Photooxidation

**DOI:** 10.1002/fsn3.4539

**Published:** 2024-11-11

**Authors:** Mahdi Hajimohammadi, Fatemeh Sheikh Mahboobi, Haizhou Wu

**Affiliations:** ^1^ Faculty of Chemistry Kharazmi University Tehran Iran; ^2^ College of Food Science and Technology Huazhong Agricultural University Wuhan China; ^3^ Department of Biology and Biological Engineering, Food and Nutrition Science Chalmers University of Technology Gothenburg Sweden

**Keywords:** erythrocyte model, flavonoid compounds, light irradiation, lipid photooxidation, oleic acid model, singlet oxygen

## Abstract

Photosensitizers and pigments in raw meat such as porphyrins, riboflavin, and myoglobin after incorporation with light beam prompt the generation of singlet oxygen (^1^O_2_) from triplet oxygen (^3^O_2_) and cause oxidative rancidity of meat products. In this study, the results of photooxidation reactions of sheep erythrocyte (red blood cell) model as a model rich in hemoglobin and phospholipids bilayer, and oleic acid model were obtained by ^1^H NMR spectroscopy, TBARS assay, and iodometric titration. In both models, the rate of lipid photooxidation in the presence of hydroalcoholic extracts of Turmeric (*Curcuma longa* L.) and Cumin (*Cuminum cyminum* L.) as natural antioxidants, Butyl hydroxytoluene (BHT) as a synthetic antioxidant, and sodium azide (NaN_3_) as a well‐known ^1^O_2_ scavenger were decreased in the order of NaN_3_ > Turmeric > Cumin > BHT. It was proven that during the photooxidation process, there is a direct association between the amount of flavonoid compounds and ^1^O_2_ scavenging.

## Graphical abstract

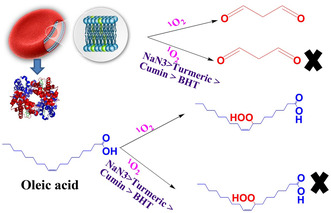

## 1 Introduction

**FIGURE 1:**
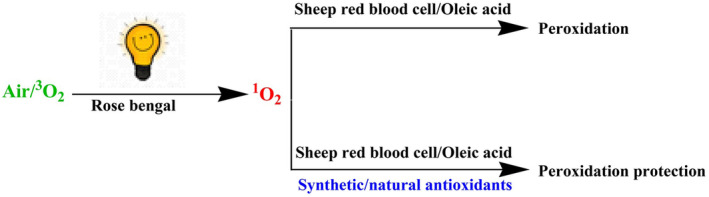
Generation or quenching of ^1^O_2_ in red blood cells (erythrocyte) and oleic acid media in the presence and absence synthetic/natural antioxidants.

Yearly, a large amount of meat products change color or flavor because of exposure to light, which incorporates an awesome effect on the sales process of meat products (Looper 2023; MacDougall 1982; Sebranek and Bacus 2007; Versino et al. 2023). Thus it is important to understand the photooxidation process and inhibition of photooxidation reactions in the muscle foods. Photosensitizers and pigments in raw meat such as porphyrins, riboflavin, and myoglobin after incorporation with light beam prompt the generation of ^1^O_2_ from ^3^O_2_ and cause oxidative rancidity of meat products. (Min and Boff 2002; Papuc et al. 2017). Due to spin rule ^3^O_2_ as a stable type of oxygen cannot react with polyunsaturated fatty acids (PUFA) but with a combination of light energy and photosensitizers, the unpaired electrons of ^3^O_2_ are paired and generate ^1^O_2_ (Martemucci et al. 2022). Electrophilic tendency of ^1^O_2_ causes to oxidize lipids, PUFA, amino acids, and electro‐rich compounds (Agnez‐Lima et al. 2012). Importantly, ^1^O_2_ can directly carry out the initiation or propagating steps of lipid oxidation in meat, whereas other reactive oxygen species (ROS) like superoxide anion radical (O_2_^−^), hydrogen peroxide (H_2_O_2_), and hydroperoxyl radical (HO_2_^·^) can be converted to more ROS using enzymes and transition metals (Domínguez et al. 2019; Droge 2002; Huang and Ahn 2019; Wu et al. 2022). After slaughtering antioxidant system loss its efficiency and in the presence of oxygen and light, meat products initiate two undesirable oxidative processes: protein oxidation and lipid peroxidation (Papuc et al. 2017; Wu, Abdollahi, and Undeland 2021). During this processes, some by‐products are formed which diminish meat quality (Papuc et al. 2017). Because of possible production of harmful and carcinogenic agents during the decomposition of synthetic antioxidants, the desire of food processing companies to use natural antioxidants over synthetic antioxidants such as butylated hydroxyanisole (BHA) and butylated toluene hydroxyl (BHT), has increased. (O'Hara et al. 1998; Wu, Sajib, and Undeland 2021). Phenolic compounds existing in natural antioxidants are one of the abundance sources of flavonoid compounds (Dumanović et al. 2021; Marcillo‐Parra et al. 2021; Shahidi et al. 2019). Generation of ^1^O_2_ with photosensitization reaction and using it for oxidation of carbon‐based compounds, DNA damage, and photodynamic therapy is a known effective method (DeRosa and Crutchley 2002; Greer 2006; Hajimohammadi and Nosrati 2018; Hajimohammadi et al. 2011, 2018), but there are lack of studies on interaction of ^1^O_2_ and muscle foods and also effect of natural antioxidant as ^1^O_2_ scavenger and their performance in the meat products preservation (Bradley and Min 1992; Ding and Chan 1984; Domínguez et al. 2019; Van Dyck 2010). The purposes of this study were as follows (1) investigation of the effect of ^1^O_2_ on erythrocyte model and oleic acid (as a fatty acid) and (2) investigation of the effect of synthetic/natural antioxidants on erythrocyte and oleic acid photooxidation (Figure 1).

## 2 Materials and Methods

### 2.1 Materials

Oleic acid, Rose Bengal, ethanol (C_2_H_5_OH), dimethyl sulfoxide (DMSO), potassium oxide (K_2_O), H_2_O_2_, and acetonitrile (CH_3_CN) were obtained from Fluka and Merck without any further purification. Turmeric and cumin methanolic extracts were obtained from Pardis Extract and Barij Essence pharmaceutical companies.

### 2.2 Preparation of Erythrocytes

Based on Dodge, Mitchell, and Hanahan (1963) method, fresh heparinized sheep blood was used for the erythrocyte membrane preparation. Finally, erythrocyte product with ca. 1 mg/mL concentration was suspended in a phosphate buffer at pH 7.4.

### 2.3 Sample Preparation for Erythrocyte Photooxidation

0.2 mL Rose Bengal (0.001 M) was added to 10 mL erythrocytes in a test tube. Then separately, 2 mL cumin extract (containing 3.62 ± 0.12 mg QE/g flavonoid), 2 mL turmeric extract (containing 4.30 ± 0.26 mg QE/g flavonoid), 0.0016 mmol BHT, and 0.0016 mmol NaN_3_ were added to the test tubes. Sample tubes were illuminated by a solar simulator (276 power LED lamps, 1 W, 2.3 V (57100 LUX), equipped with cooling fan) for 6 h at 25°C under 1 atm air bubble.

### 2.4 Sample Preparation for Oleic Acid Photooxidation

Two milliliters of cumin extract (containing 3.62 ± 0.12 mg QE/g flavonoid), 2 mL turmeric extract (containing 4.30 ± 0.26 mg QE/g flavonoid), 0.0016 mmol BHT, and 0.0016 mmol NaN_3_ separately were added to solution of oleic acid (4.6 × 10^−3^ M) and Rose Bengal (1 × 10^−3^ M). Sample tubes were illuminated by a solar simulator (276 power LED lamps, 1 W, 2.3 V [57100 LUX], equipped with cooling fan) for 120 min at 25°C under 1 atm air bubble.

### 2.5 Analytical Methods

Based on malonic dialdehyde released in the samples, a thiobarbituric reactive substances (TBARS) assay was applied to obtain lipid oxidation in erythrocyte by UV–Vis spectroscopy (Shimadzu 2100 spectrophotometer) at 532–535 nm (Kleszczyńska et al. 1998). The peroxide value (PV, meq O_2_/kg) of the samples was measured according to the Barthel and Grosch (1974) method. Proton nuclear magnetic resonance (^1^H NMR) spectra were recorded on a Bruker AMX 300 MHz spectrometer using TMS as an internal standard.

### 2.6 Statistical Analysis

**FIGURE 2:**
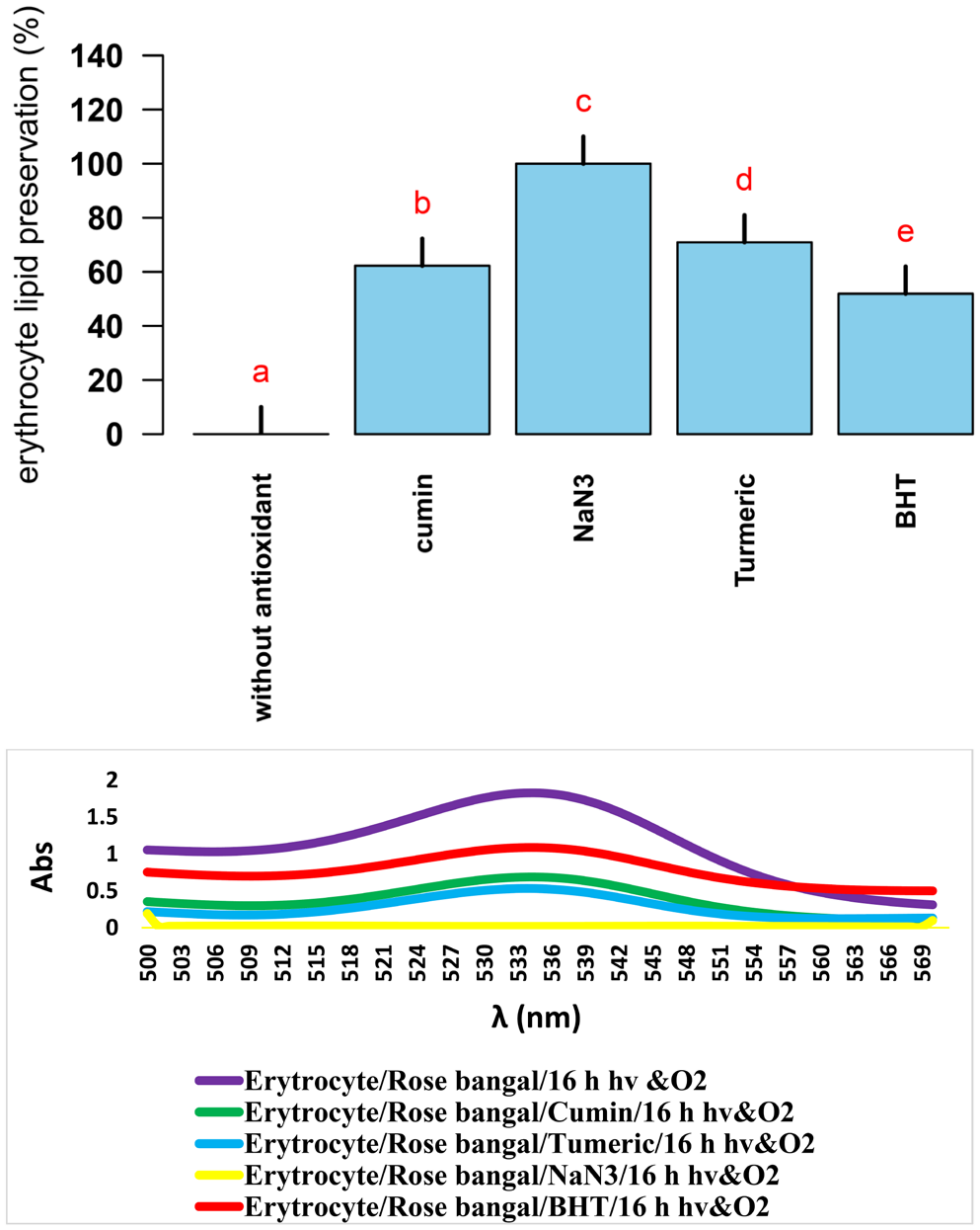
Preservation of lipids of sheep erythrocyte from photooxidation in the presence of NaN_3_, turmeric, cumin, and BHT. Different lowercase letters (from the highest *p* value to the lowest *p* value [a–e]) indicate statistically significant differences according to Duncan's test (*p* < 0.05).

All experiments used three replicates and results were analyzed with SAS software version 3.9. Results were then averaged and compared using Duncan's test. R statistical package was used to plot the graphs. Results were presented as mean ± standard deviation (SD) (*n* ≥ 2). The significance threshold for all experiments was set at *p* < 0.05. To test the effect of antioxidants and obtaining lipids preservation percentages in sheep erythrocyte model, the average TBARS values of three replicates of erythrocyte photooxidation reactions in the presence of NaN_3_, turmeric, cumin, and BHT were compared with TBARS value obtained under nonantioxidant reaction condition (Figure 2). According to the Duncan post hoc tests, all experimental groups showed statistically significant differences with each other and with the control group (without antioxidant) (*p* < 0.05). Nonsignificant values (*p* > 0.05) were excluded in a stepwise manner. Also, to test the effect of antioxidants and obtain oleic acid preservation percentages, the average PVs of three replicates of photooxidation reactions of oleic acid in the presence of NaN_3_, turmeric, cumin, and BHT were compared with PV obtained under nonantioxidant reaction condition (Figure 4). According to the Duncan post hoc tests, all experimental groups showed statistically significant differences with each other and with the control group (without antioxidant) (*p* < 0.05). Nonsignificant values (*p* > 0.05) were excluded in a stepwise manner.

## 3 Results and Discussion

### 3.1 Evidences for Singlet Oxygen Generation in the Photooxidation of Oleic Acid and Sheep Erythrocyte Model

**FIGURE 3:**
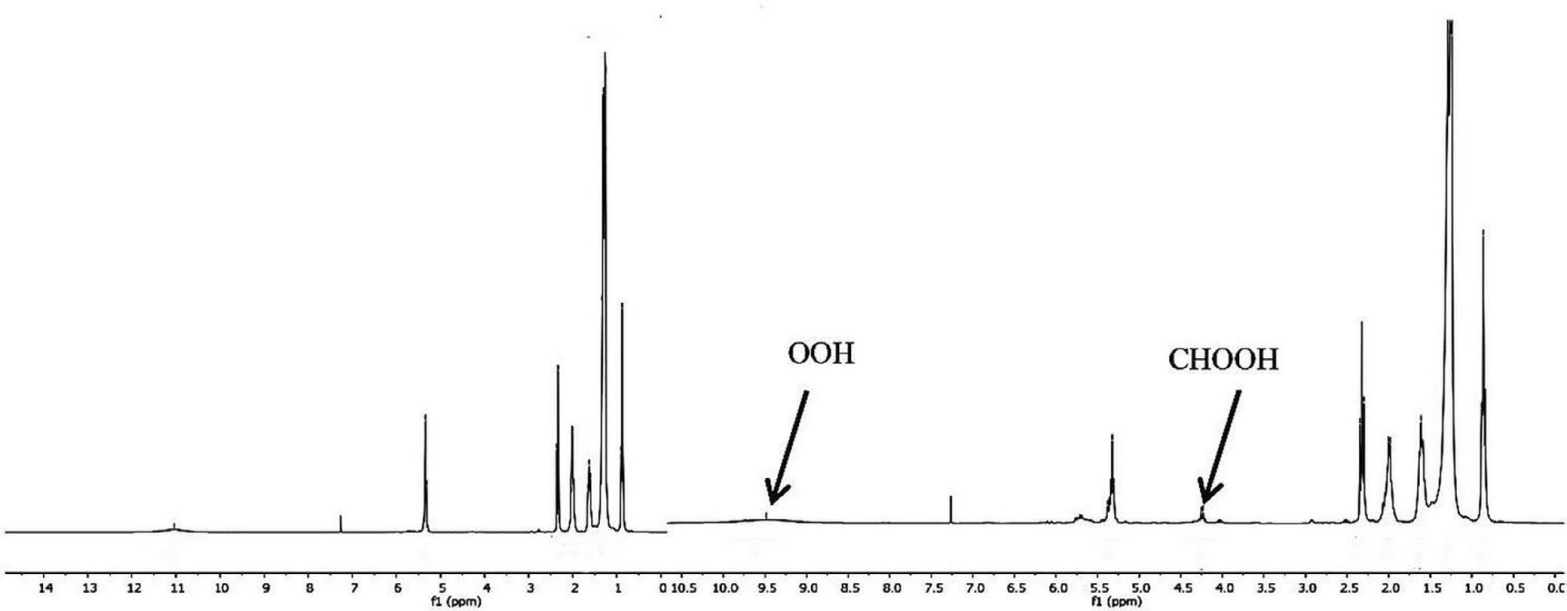
^1^H NMR spectra of oleic acid (4.6 × 10^−3^ M) after photooxidation with photosensitizer (right) and without photosensitizer (left).

As a typical standard reaction for evaluating ^1^O_2_ generation, photooxidation of oleic acid was investigated using Rose Bengal as a photosensitizer. Photooxidation of oleic acid was accomplished by ^1^H NMR spectroscopy and iodometric method. Results of ^1^H NMR spectroscopy (Figure 3) and iodometric method declared that in the absence of Rose Bengal, light or oxygen formation of peroxide products from oleic acid was stopped (Table 1, Entry 1–3). Therefore, the existence of a photosensitizer, light, and O_2_ is indispensable for the photooxidation of oleic acid to corresponding products. Also, when N_3_^−^ as a standard of ^1^O_2_ quenching (Lolak et al. 2022) was applied, the photooxidation conversion of oleic acid and photodegradation of the Rose Bengal was diminished (Table 1, Entry 5). It was interesting that lipid oxidation in erythrocyte media in the presence of NaN_3_ significantly was reduced, which prove ^1^O_2_ generation (Table 1, Entries 6 and 7). Two main mechanisms are reported for photooxidation reactions with nonmetallic photosensitizers, Type I and Type II (Figure 6a) (Huang et al. 2020). Reaction of substrates with ^1^O_2_ is the primary pathway that occurs in our reaction conditions (Type II). Table 1 Entries 4 and 8 show that the formation of peroxide products from oleic acid in acetonitrile solvent is higher than ethanol solvent. These results are correlated with ^1^O_2_ lifetime in acetonitrile (65 μs) and ethanol (38 μs) solvents (Bressan and Morvillo 1989; Chen et al. 2001; Toffoli et al. 2008). In addition to these results, trace formation of peroxide products in the presence of O_2_^−^, indicating that the dominant pathway under our reaction conditions is not the Type I mechanism. (Table 1, Entry 9).

**TABLE 1:** Photooxidation of oleic acid and erythrocyte by ^1^O_2_ under various reaction conditions^a^.

Entry	Reaction condition	Oleic acid conversion (%)
1	Oleic acid + CH_3_CN + air + light	Trace
2	Oleic acid + CH_3_CN + Rose Bengal + air	Trace
3	Oleic acid + CH_3_CN + Rose Bengal	Trace
4	Oleic acid + CH_3_CN + Rose Bengal + light + air	100
5^b^	Oleic acid + CH_3_CN + Rose Bengal + NaN_3_ + light + air	Trace
6	Erythrocyte + CH_3_CN + Rose Bengal + NaN_3_ + light + air	100
7^b^	Erythrocyte + CH_3_CN + Rose Bengal + NaN_3_ + light + air	Trace
8	Oleic acid + C_2_H_5_OH + Rose Bengal + light + air	35
9^c^	Oleic acid + O_2_^−^	Trace

^a^ Solution of oleic acid (4.6 × 10^−3^ M) or erythrocyte and Rose Bengal (1 × 10^−3^ M) was illuminated by a solar simulator (276 power LED lamps, 1 W, 2.3 V (57100 LUX), equipped with cooling fan) for 120 min at 25°C under 1 atm air bubble.

^b^ 0.0016 mmol NaN_3_ was used.

^c^ O_2_^−^ was produced by dissolving potassium oxide in dried DMSO (Sawyer 1991).

### 3.2 Effect of Turmeric and Cumin on Sheep Erythrocyte Model Photooxidation

In this study, the oxidative reactions in sheep red blood cells resulting from oxidation by ^1^O_2_ in the presence and absence of synthetic/natural antioxidants were investigated (Figure 2). The reason for choosing erythrocyte as a model for studying lipid oxidation in muscles is that the remaining blood accelerates the lipid oxidation of the phospholipid bilayer by hemolyzing and releasing hemoglobin. (Richards and Hultin 2002). Consequently, the capacity of red blood cells to withstand oxidative stress and remain intact is important for muscle oxidative stability. The effect of ^1^O_2_ on lipid oxidation in erythrocyte media in the presence of cumin, turmeric as natural antioxidants (Ali et al. 2021), NaN_3_ as a strong ^1^O_2_ scavenger (Lolak et al. 2022), and BHT as a highly effective synthetic antioxidant (Yehye et al. 2015) were investigated (Figure 2). The antioxidant results showed that NaN_3_, turmeric, cumin, and then BHT were able to prevent the photooxidation conversion of lipids into peroxide products in sheep erythrocyte model by 100%, 70.93%, 62.26%, and 51.92%, respectively.

### 3.3 Effect of Turmeric and Cumin on Oleic Acid Photooxidation

**FIGURE 4:**
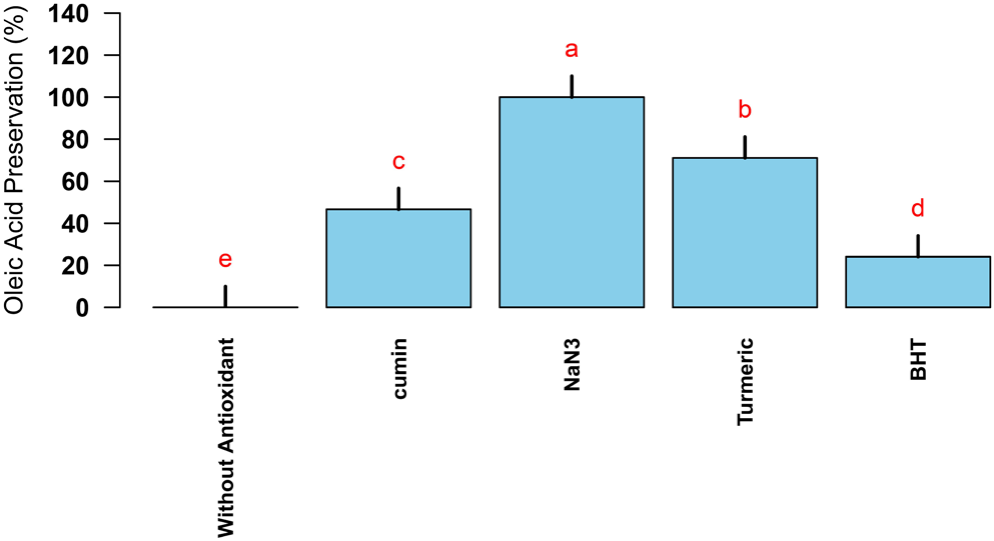
Preservation of oleic acid from photooxidation in the presence of NaN_3_, turmeric, cumin, and BHT. Different lowercase letters (from the highest *p* value to the lowest *p* value [a–e]) indicate statistically significant differences according to Duncan's test (*p* < 0.05).

The photosensitized production of singlet oxygen has significance in the areas of the photooxidation of organic compounds and food chemistry (Domínguez et al. 2019; Hajimohammadi and Nosrati 2018; Hajimohammadi et al. 2018). Photooxidation of oleic acid as one of the targets of singlet oxygen was investigated to evaluate the antioxidant effect of turmeric and cumin. Inhibition values in oleic acid conversion to peroxide products in the presence of NaN_3_, turmeric, cumin, and BHT were obtained by 100%, 71.10%, 46.63%, and 24.08%, respectively (Figure 4).

### 3.4 Discussion

**FIGURE 5:**
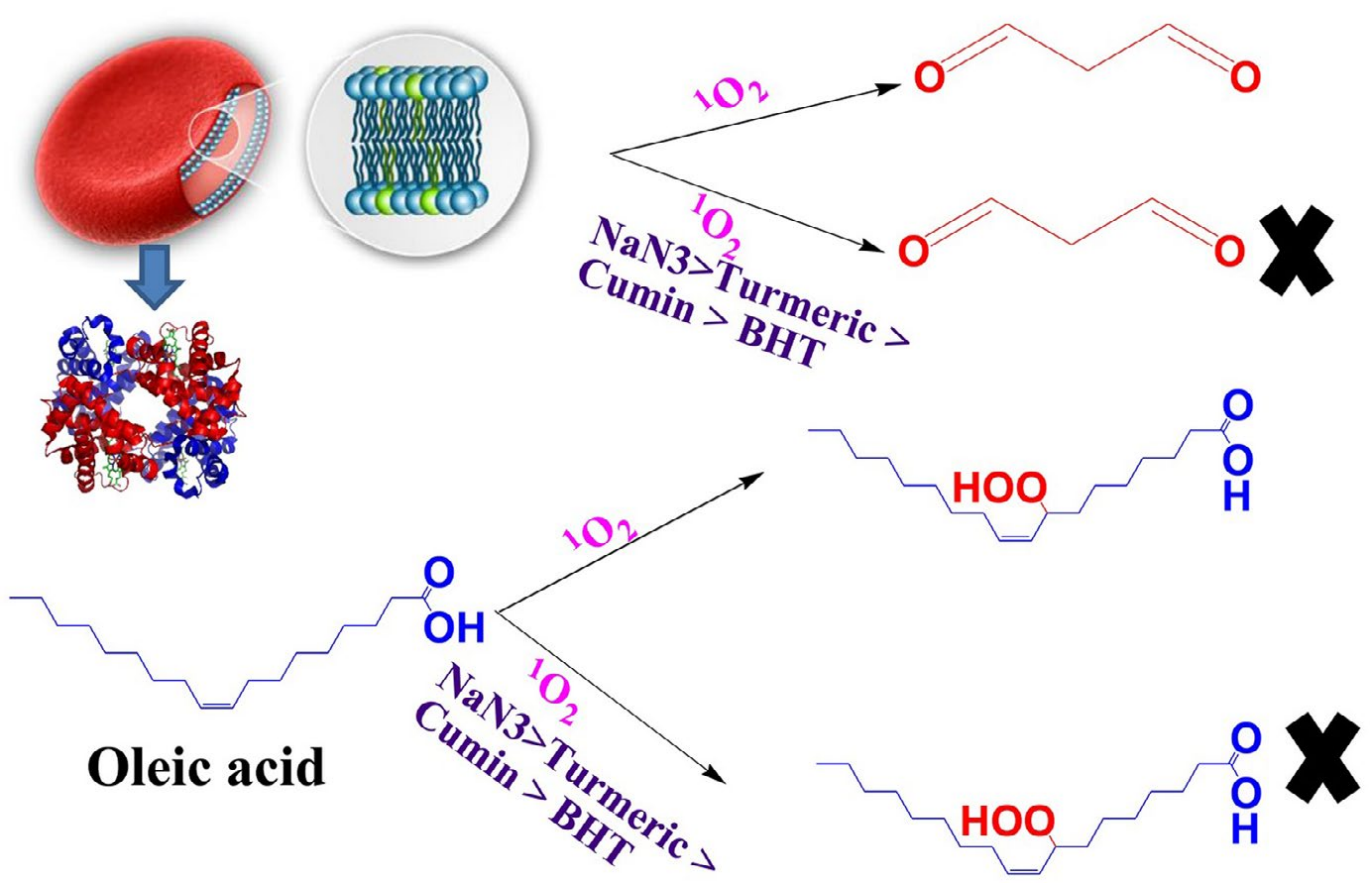
Suppressing effect of plants contain flavonoid compounds on lipids photooxidation of sheep red blood cells and oleic acid photooxidation.

**FIGURE 6:**
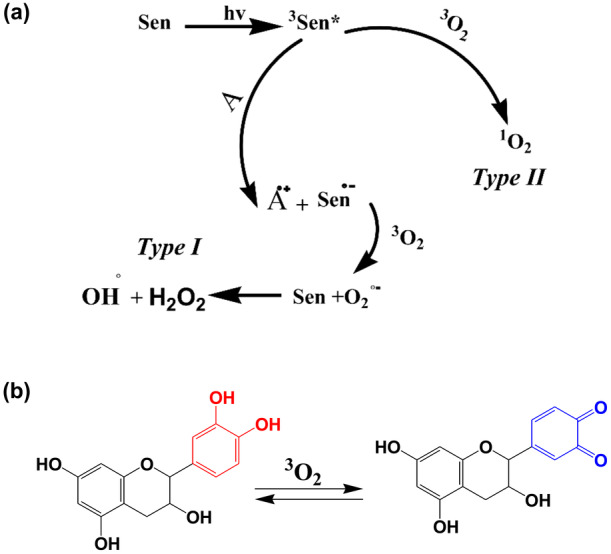
Pathways of ROS generation in photooxidation process (a), Mechanism of ^1^O_2_ quenching by flavonoid compounds (b).

In biological systems, along with photosensitization rout of ^1^O_2_ generation, H_2_O_2_ can react with superoxide anions or with HOCl or chloramines to form ^1^O_2_ (Vašková, Vaško, and Kron 2012). Nonenzymatic lipid peroxidation is detected by an arachidonic acyl group, and the start of chain reaction is explained by three pathways: ^1^O_2_, hydroxyl radical generation from the Fenton reaction, and perferryl‐myoglobin. ^1^O_2_ can directly accomplish the initiation or propagation stages of lipid oxidation, whereas O_2_^˙−^, H_2_O_2_, and hydroperoxyl radical HO_2_^˙^ can be converted to more ROS using enzymes and transition metals (Papuc et al. 2017). In this study, the oxidative reactions in sheep red blood cells and oleic acid resulting from oxidation by ^1^O_2_ in the presence and absence of synthetic/natural antioxidants were investigated (Figure 5). Several studies have mentioned the high amount of flavonoid compounds in cumin and turmeric (Ali et al. 2021; Yashin et al. 2017). The results of this study declared that the two methods have a respectable match for investigating turmeric and cumin as a source of flavonoid compounds on photooxidation of lipids and fatty acids with ^1^O_2_. Interestingly, the rates of oleic acid oxidation and lipid oxidation in the erythrocyte model decreased in the order of turmeric > cumin in the presence of natural antioxidants that correlate with the amount of flavonoid compounds in turmeric (containing 4.30 ± 0.26 mg QE/g flavonoid) and cumin (containing 3.62 ± 0.12 mg QE/g flavonoid). According to the literature, plant and natural sources of flavonoid and polyphenolic compounds have been acting as an inhibitor of ROS (Mitra 2018). Flavonoid compounds because of a strong tendency to ^1^O_2_, in contact with ^1^O_2_ readily oxidized and generate quinone products (Mitra 2018). (Figure 6b).

## 4 Conclusion

Photooxidation of lipids with ^1^O_2_ is an undesirable chemical process in which unsaturated fatty acids are converted into peroxides, and consequently causes oxidative rancidity of meat products. Therefore, scavenging of ^1^O_2_ is vital to maintain meat quality. Antioxidants play an important role in preventing the oxidation of biomolecules by inhibiting the radical chain reaction, but after slaughtering their effectiveness is lost when the antioxidant system is disrupted. This study was aimed at investigation of combination of light and molecular oxygen for lipid photooxidation in sheep erythrocyte model and oleic acid model in the presence of synthetic and natural antioxidants. In this study, ^1^O_2_ production in erythrocyte model and oleic acid medium as a fatty acid model was proved. Also, the higher antioxidant capacities of cumin and turmeric as natural antioxidants against ^1^O_2_, in comparison with BHT as a synthetic antioxidant were verified. It was found that the rate of ^1^O_2_ quenching is connected to the amount of flavonoid compounds. It seems that doping flavonoid compounds to meat products has a significant effect on maintaining the quality of meat and, as a result, the sales process of meat products. Further studies should be done toward the finding of new natural antioxidants in order to improve meat and meat product preservation.

## Author Contributions

**Mahdi Hajimohammadi:** conceptualization (lead), data curation (lead), formal analysis (lead), funding acquisition (lead), investigation (lead), methodology (lead), project administration (lead), resources (lead), software (lead), supervision (lead), validation (lead), visualization (lead), writing – original draft (lead), writing – review and editing (lead). **Fatemeh Sheikh Mahboobi:** conceptualization (equal), formal analysis (equal), investigation (equal), software (equal). **Haizhou Wu:** writing – review and editing (equal).

## Ethics Statement

The authors have nothing to report.

## Conflicts of Interest

The authors declare no conflicts of interest.

## Data Availability

The data are available upon request from the authors.
